# The *TLR9* 2848C/T Polymorphism Is Associated with the CMV DNAemia among HIV/CMV Co-Infected Patients

**DOI:** 10.3390/cells10092360

**Published:** 2021-09-08

**Authors:** Agnieszka Jabłońska, Elżbieta Jabłonowska, Mirosława Studzińska, Juliusz Kamerys, Edyta Paradowska

**Affiliations:** 1 Laboratory of Virology, Institute of Medical Biology, Polish Academy of Sciences, 93-232 Lodz, Poland; a.jablonska@ug.edu.pl (A.J.); studmir@gmail.com (M.S.); 2 Department of Infectious Diseases and Hepatology, Medical University of Lodz, 91-347 Lodz, Poland; elzbieta.jablonowska@umed.lodz.pl (E.J.); juliusz.kamerys@umed.lodz.pl (J.K.)

**Keywords:** cytomegalovirus, human immunodeficiency virus, single nucleotide polymorphism, toll-like receptor

## Abstract

Toll-like receptors (TLRs) recognize pathogen-associated molecular patterns and are essential components of the host’s innate immune response. The aim of this study was to determine the *TLR9* genotype frequency and investigate the association between *TLR9* polymorphisms and cytomegalovirus (CMV) DNAemia in human immunodeficiency virus (HIV)/CMV co-infected patients. A total of 205 HIV/CMV co-infected adults were screened for the presence of the four *TLR9* polymorphisms (−1237T/C, −1486T/C, 1174G/A, and 2848C/T) by using polymerase chain reaction-restriction fragment length polymorphism (PCR-RFLP). Mutation presented in at least one allele of the *TLR9* 2848C/T single nucleotide polymorphism (SNP) was associated with the occurrence of CMV DNAemia among HIV-infected patients with CMV co-infection (*p* = 0.004). The level of CMV DNA was higher in patients who were homozygous recessive or heterozygous for the 2848C/T polymorphism compared with those who had a wild-type genotype for this polymorphism (*p* = 0.005). Mutation detected in at least one allele of this SNP was also associated with a lower interferon type β (IFN-β) concentration (*p* = 0.048), while no relationships between *TLR9* −1237T/C, −1486T/C, and 1174G/A SNPs and CMV DNAemia were observed. Our findings suggest that the mutation present in at least one allele of the *TLR9* 2848C/T SNP may be associated with the active CMV infection in HIV/CMV co-infected subjects.

## 1. Introduction

Human cytomegalovirus (CMV) is a member of the *Herpesviridae* family and is a leading cause of morbidity and mortality in immunocompromised hosts, including patients with AIDS, organ transplant recipients, and neonates [1,2,3]. The majority of CMV infections are asymptomatic or subclinical among immunocompetent hosts. CMV infection in immunocompromised patients may lead to various clinical manifestations, such as pneumonia, nephritis, hepatitis, gastroenteritis, or central nervous system disease of severe or even fatal course [4,5]. Furthermore, CMV retinitis is the most disabling and characteristic disease in AIDS patients [4]. Hence, the identification of host molecular determinants of the innate immune response to viruses is critical to understanding the pathogenesis of HIV/CMV co-infection and developing novel therapeutic strategies.

Toll-like receptors (TLRs) are a family of transmembrane proteins, which are essential components of the innate immune response to various bacterial, parasitic, and viral pathogens. TLRs recognize and bind conserved pathogen-associated molecular patterns (PAMPs) shared by large groups of microorganisms, which leads to the production of type I interferons (IFNs) and other proinflammatory cytokines. TLR9 recognizes cytosine-phosphate-guanosine (CpG) motifs in viral DNA and activates interferon regulatory factor 7 (IRF-7), which leads to type I IFNs secretion. Host-specific differences in the *TLR9* gene are likely to alter the course of an adaptive immune response, especially in chronic viral infection [6,7,8]. It was found that chronic viral infections can impair TLR9 signaling and down-regulate TLR9 gene expression [9]. Several published studies have analyzed the role of *TLR* single nucleotide polymorphisms (SNPs) in immunocompromised patients with CMV infection [10,11,12,13,14,15,16,17,18]. A large genome-wide association cohort study in patients after hematopoietic cell transplantation revealed that the *TLR9* SNPs were associated with the CMV reactivation (rs5743836) and disease (rs352140) [11]. The *TLR9* rs352139 and rs352140 SNPs had a significant impact on the risk of acute graft-versus-host disease and early CMV infection in allogeneic hematopoietic recipients [12]. The findings suggest a potentially important role of host *TLR9* polymorphisms in HIV disease progression [8,19,20,21,22,23,24,25,26,27,28]. The *TLR9* rs352140 GG genotype was associated with an increased risk of HIV infection, disease progression, and CD4 count [20,27,29]. Mutation present in at least one allele of the *TLR9* 1635A/G SNP (rs352140) was found to be associated with increased CMV acquisition in HIV-exposed infants [19]. Two *TLR9* polymorphisms, −1237T/C (rs5743836) and −1486T/C (rs187084), are located within the promoter region and most likely alter the functional ability of the receptor. Functional analysis of the −1237T/C SNP showed that the C allele created a putative NF-kB binding site and increased *TLR9* transcriptional activity, driven mainly by activation of NF-kB [30]. Although the 2848G/A SNP (rs352140) is a synonymous substitution, it was associated with alterations in gene expression [31].

The *TLR4* Asp299Gly and Thr399Ile polymorphisms have also influenced the susceptibility of other serious infections (e.g., CMV and toxoplasmosis) in patients with advanced HIV infection [32]. However, studies focusing on the effect of *TLR9* SNPs on HIV/CMV co-infection and CMV replication in adult subjects are lacking.

In the present study, we elucidated the *TLR9* genotype distribution and investigated the correlation between the four specific *TLR9* SNPs (−1237T/C, −1486T/C, 1174G/A, and 2848C/T) and CMV DNAemia in HIV/CMV co-infected subjects.

## 2. Materials and Methods

### 2.1. Study Population

The study was approved by the Ethics Committee of the Medical University of Lodz (RNN/211/06/KE and RNN/33/17/KE) and was conducted in accordance with the Declaration of Helsinki and the good clinical practice guidelines. Written informed consent was obtained from all participants and parents/legal guardians of participants (age < 18 years) before study entry. DNA isolated from the whole blood of 205 HIV/CMV co-infected patients (median age: 37.2; range 17–72 years) were included in this study (Table 1). From 2006 to 2008, and from May 2017 to March 2018, 43 HIV/CMV co-infected subjects with a CD4^+^ T count ≤200 cells/mm^3^ and 162 individuals with a CD4^+^ T cell count > 200 cells/mm^3^ were bled once for this study. The HIV/CMV co-infected subjects were enrolled in this study during routine HIV disease monitoring. The HIV RNA was detected in 35/205 (17.1%) subjects. The mean HIV RNA concentration in peripheral blood was 3.98 × 10^3^ copies/mL ± 4.11 × 10^4^ copies/mL (median: 0, range: 0–5.82 × 10^5^ copies/mL). HIV viremia was determined using the RT-PCR method (Cobas AmpliPrep/Cobas TaqMan HIV-1 Test, Roche Diagnostics GmbH, Mannheim, Germany). CMV infection was confirmed by the detection of CMV DNA in the whole blood and/or specific anti-CMV IgG antibodies in the serum samples. The CMV IgG antibodies were detected in 192/205 (93.7%) of patients with HIV/CMV co-infection and undetected in 13/205 (6.3%) of subjects. Patients with negative serologic status were positive for the CMV DNAemia. No symptoms specific for cytomegaly were observed in almost all (203/205, 99.0%) patients with HIV/CMV co-infection. Among patients who developed symptoms, one patient had retinitis, while pneumonia has been reported in the second patient. All patients were Caucasian and were enrolled from the central area of Poland (Lodz).

### 2.2. Detection of TLR9 Polymorphisms

Genomic DNA was isolated from blood samples using a QIAamp DNA Blood Mini Kit (Qiagen GmbH, Hilden, Germany) according to the manufacturer’s instructions. Genotyping for *TLR9* (−1237T/C, rs5743836; −1486T/C, rs187084; 1174G/A, rs352139; and 2848C/T, rs352140) SNPs were performed by polymerase chain reaction-restriction fragment length polymorphism (PCR-RFLP) as we described elsewhere [10]. The reaction was performed in a Veriti 96 Well Thermal Cycler (Applied Biosystems, Foster City, CA, USA). The concentration and purity of DNA were determined using a NanoDrop 2000c UV-Vis Spectrophotometer (Thermo Scientific, Waltham, MA, USA). The digested fragments were analyzed using a QIAxcel system (Qiagen). The selected samples of each *TLR9* SNP were sequenced using the 96-capillary 3730xl DNA Analyzer (Applied Biosystems) to confirm the detected genotypes.

### 2.3. Assessment of CMV DNAemia

The analyses of the CMV DNA copy number in DNA isolates from the whole blood samples were performed using quantitative real-time PCR (qRT-PCR) as previously described [33]. The amplification was performed using a 7900HT Fast Real-Time PCR system (Applied Biosystems) with the primer set located within the *UL55* gene.

### 2.4. Measurement of Cytokine Levels

The concentration of serum cytokines was determined using the BD Cytometric Bead Array (CBA) Human Th1/Th2/Th17 Cytokine kit (BD Biosciences, San Jose, CA, USA) by the LSR II BD flow cytometer (BD Biosciences, Franklin Lakes, NJ, USA). Levels of IFN-α, IFN-β (PBL Assay Science, Piscataway, NJ, USA), and IL-7 (Thermo Scientific, Frederick, MD, USA) were estimated using ELISA kits according to the manufacturer’s recommendations.

### 2.5. Statistical Analyses

Statistical analyses were performed using GraphPad Prism 5.00 (GraphPad Software, San Diego, CA, USA) or SPSS 25.0 for Windows (SPSS Inc., Chicago, IL, USA). The comparison of genotypes and the allele frequency between the HIV/CMV co-infected patients with or without CMV DNAemia was evaluated by Fisher’s exact test. The Mann-Whitney U test was used to describe the relationship between the *TLR9* SNPs and CMV DNA copy number or IFN-β concentration. Hardy-Weinberg equilibrium (HWE), linkage disequilibrium (LD), and haplotype analyses were performed using the SNPStats software (http://www.snpstats.net/start.htm, accessed on 30 November 2020). The odds ratio (OR) with a 95% confidence interval (95% CI) for assessing the effect of genotype distribution and allelic frequencies on the occurrence of CMV DNAemia in co-infected subjects was calculated by logistic regression analysis. Statistical significance was set at *p* ≤ 0.05. Bonferroni’s correction of the significance level (*p^c^*) was applied to account for multiple testing. The *p^c^*-value was count by dividing the *p*-value by the number of comparisons (the total number of SNPs in the data set, *n* = 3). The level of significance was *p^c^* < 0.017 (0.05/3).

## 3. Results

### 3.1. Heterozygous and Homozygous Recessive Genotypes of the TLR9 2848C/T SNP Are Prevalent in HIV/CMV Co-Infected Patients with CMV DNAemia

The *TLR9* −1237T/C, −1486T/C, 1174G/A, and 2848C/T SNPs were genotyped in 205 HIV/CMV co-infected patients. The demographic and clinical characteristics of the examined patients are summarized in Table 1. The wild-type −1237 TT genotype was detected in almost all (199/205; 97.1%) of HIV/CMV co-infected patients (Table 2). No significant differences for −1486C/T and 1174G/A SNPs were found. For the *TLR9* 2848C/T SNP, the genotype distribution was significantly different between the HIV/CMV co-infected patients with CMV DNAemia and in co-infected subjects without CMV DNA in blood samples. Mutation present in at least one allele of the 2848C/T SNP was detected more frequently in HIV/CMV co-infected patients with CMV DNAemia than in co-infected individuals without CMV DNAemia (76.0% vs. 57.2%; *p* = 0.006). The observed genotype frequency of the *TLR9* −1486T/C SNP was not in HWE among HIV/CMV co-infected patients and was excluded from further analysis (*p* > 0.05).

Considering the distribution of *TLR9* alleles, no significant differences in the frequency of −1237T/C and 1174G/A alleles were observed (Table 2, *p* > 0.05). In the case of the 2848C/T SNP, the T allele was more common in HIV/CMV co-infected patients with CMV DNAemia than in subjects without CMV DNA in the blood (52.1% vs. 37.5%, *p* = 0.005; Table 2).

### 3.2. Mutation Present in at Least One Allele of the TLR9 2848C/T SNP Occurs More Frequently in HIV/CMV Co-Infected Patients with CMV Viremia

Mutation presented in at least one allele of the *TLR9* 2848C/T SNP occurred at least two times more frequently in HIV/CMV co-infected patients with CMV DNAemia than in HIV/CMV co-infected subjects without CMV DNA (OR: 2.38; 95% CI: 1.30–4.34; *p* = 0.004, in the dominant model; Table 3). This *TLR9* SNP also showed a higher rate of occurrence of viral co-infection even after Bonferroni correction for multiple testing (*p^c^* = 0.017) in the unadjusted model (Table 3). A statistically significant difference was noticed for the influence of the heterozygous genotype of the *TLR9* 2848C/T SNP on the CMV DNAemia among individuals undergoing antiretroviral treatment (OR: 2.44; 95% CI: 1.25–4.76; *p* = 0.0049, in the codominant model). In addition, the TT genotype of the *TLR9* 2848C/T SNP was more frequently observed in the HIV/CMV co-infected individuals with the CD4^+^ T cell count ≤200 cells/mm^3^ than in HIV/CMV co-infected patients with the CD4^+^ T cell count >200 cells/mm^3^ (OR: 2.96; 95% CI: 1.23–7.12; *p* < 0.001).

### 3.3. The TLR9 2848C/T Polymorphism Is Associated with the CMV Viremia

**Figure 1:**
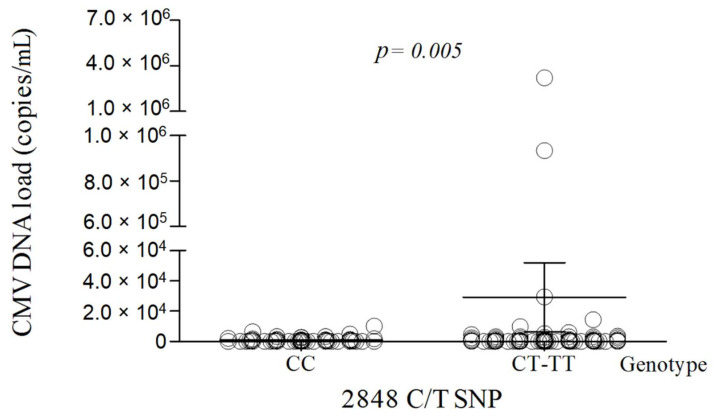
Comparison of the CMV DNAemia level in HIV/CMV co-infected subjects with or without the specific *TLR9* 2848C/T SNP (*n* = 205). Bars in the scatter dot plot represent the mean viral loads and whiskers represent the standard error of the mean (SEM) values. *p*-value via a Mann–Whitney U test.

Of the 205 patients with HIV/CMV co-infection, 121/205 (59.0%) had detectable CMV DNAemia levels in the peripheral blood (mean: 2.12 × 10^4^ ± 2.34 × 10^5^ copies/mL; range: 0–3.22 × 10^6^ copies/mL). Among patients with CMV retinitis or pneumonia, a moderate CMV DNAemia level was noticed (1.44 × 10^4^ copies/mL and 2.93 × 10^4^ copies/mL, respectively). To further examine the association between the *TLR9* and CMV, we correlated the polymorphisms with the degree of CMV DNAemia. As shown in Figure 1, the mean level of CMV DNA was higher among individuals who were homozygous recessive or heterozygous for the *TLR9* 2848C/T SNP (mean 2.91 × 10^4^ ± 2.76 × 10^5^ copies/mL) compared with those who had a wild-type genotype for this polymorphism (mean 9.83 × 10^2^ ± 1.75 × 10^3^ copies/mL; *p* = 0.005; Mann-Whitney U test). The patients with heterozygous and homozygous recessive genotypes at the 2848 locus had almost three-fold increased risk of high CMV DNA copy numbers (>10^3^ copies/mL) (OR: 2.863; 95% CI: 1.524–5.379; *p* = 0.001). No association was observed between the CMV DNAemia and *TLR9* −1237T/C and 1174G/A SNPs (*p* > 0.05). The mean level of CMV DNAemia was higher in HIV/CMV co-infected patients with CD4^+^ T count ≤ 200 cells/mm^3^ (mean: 9.63 × 10^4^ ± 5.02 × 10^5^ copies/mL; range: 0–3.22 × 10^6^ copies/mL) compared with those who had CD4^+^ T count > 200 cells/mm^3^ (mean: 6.41 × 10^2^ ± 1.31 × 10^3^ copies/mL; range: 0–1.02 × 10^4^ copies/mL, respectively; *p* = 0.041). No other association with CD4^+^ T cell count, age, and CMV DNAemia was observed (*p* > 0.05).

### 3.4. The TGT Haplotype of TLR9 −1237T/C, 1174G/A, and 2848C/T SNPs Is Prevalent in HIV/CMV Co-Infected Patients

Haplotype analysis of *TLR9* −1237T/C, 1174G/A, and 2848C/T SNPs showed that the most frequent haplotype was TGT (30.4% and 42.9% for HIV/CMV co-infected patients with or without CMV DNAemia, respectively). The next most prevalent haplotype was TGC (34.1% and 21.4% for HIV/CMV co-infected patients with CMV DNAemia and HIV/CMV co-infected subjects without CMV DNAemia, respectively) and was associated with a higher rate of the occurrence of CMV viremia (OR: 1.95; 95% CI: 1.10–3.47; *p* = 0.023). No evidence of LD in −1237T/C, −1174G/A, and 2848C/T SNPs was found (r^2^ < 0.2).

### 3.5. The TLR9 2848C/T SNP Is Associated with Lower Serum IFN-β Level

**Figure 2:**
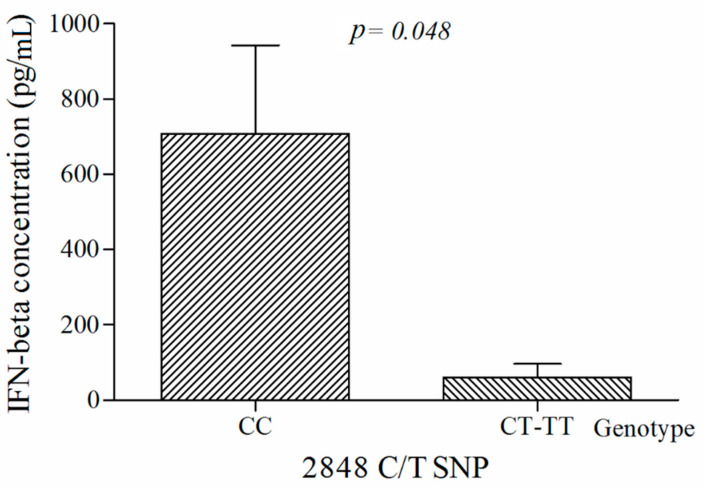
Interferon-β (IFN-β) levels by *TLR9* 2848C/T SNP in HIV/CMV co-infected patients. Bars represent the mean values and standard error of the mean (mean ± SEM) of the IFN-β concentration. *p* < 0.05 via a Mann-Whitney U test.

To further investigate the clinical relevance of *TLR9* SNPs and the outcome of infection, we determined the serum concentrations of target cytokines in HIV/CMV co-infected patients. Subjects who had wild-type genotype for the *TLR9* 2848C/T SNP had higher serum IFN-β concentration compared with those who were heterozygous or homozygous recessive for this polymorphism (708.2 pg/mL ± 1126.7 pg/mL vs. 60.7 pg/mL ± 128.0 pg/mL; *p* = 0.048; Figure 2). No other cytokines showed significant associations with the *TLR9* SNPs in both of the study groups.

## 4. Discussion

It was found that the common polymorphisms of various *TLR* genes may be associated with increased susceptibility to or protection from several infections [20,21,32,34,35,36]. Moreover, the genetic variability in TLR signaling pathway molecules (i.e., interleukin-1 receptor-associated kinase 4 [IRAK4], an inhibitor of nuclear factor κB kinase γ [IKKγ], and an inhibitor of nuclear factor κB α [IκBα]) causes rare inherited immunodeficiencies that can also influence the susceptibility to human diseases [37,38]. Among the TLRs, intracellular TLR9 recognizes unmethylated cytosine-phosphate-guanosine (CpG) DNA motifs in bacteria and viruses [39,40] and is critically required in the process of CMV sensing [41,42]. TLR9 is mainly expressed by cells of the immune system such as dendritic cells, monocytes/macrophages, natural killer cells, and lymphocytes [43]. TLR9 triggers signaling cascades that lead to a pro-inflammatory cytokine response, while TLR ligands induce immune activation in vitro in CD4^+^ and CD8^+^ T lymphocytes derived from HIV patients [44]. Host-specific differences in the *TLR9* gene are likely to alter the course of an adaptive immune response, especially in a chronic infection like HIV or CMV. To date, the *TLR9* SNPs have been showing to modulate the risk of CMV infection in individuals with the allogeneic hematopoietic stem cell transplantation (allo-HSCT) [11,12], renal transplant recipients [13,14], and infants [10,15]. The *TLR9* 2848C/T SNP was associated with congenital CMV infection in fetuses and infants [10,15]. Moreover, the *TLR9* 1635 G (rs352140) allele has been associated with HIV load and disease progression [20,21].

In the present study, we report, for the first time, a relationship between the polymorphisms of the *TLR9* gene and the incidence of CMV DNAemia among HIV/CMV co-infected subjects. The mutation detected in at least one allele of the *TLR9* 2848C/T SNP occurred more frequently in HIV patients with CMV viremia than in subjects without CMV DNAemia. The *TLR9* 2848 TT genotype seems to be associated with higher levels of CMV DNAemia and CD4^+^ T cell depletion, whereas the subjects who had a wild-type genotype for this SNP had higher serum IFN-β concentration. Interestingly, the results of other studies have shown that two common *TLR9* polymorphisms, −1486C/T and 1635A/G, may be associated with disease progression in HIV-infected patients [20,22]. Joshi et al. demonstrated an association between *TLR9* 1635A/G SNP and HIV mediated immune activation in both CD4^+^ and CD8^+^ T cells as well as interferon gamma-induced protein 10 (IP10) levels [8]. The *TLR9* 1635 G allele reduced HIV acquisition [23], was associated with lower HIV load [21], and was protective against disease progression in HIV-infected adults [21,26]. Most notably, the *TLR9* 1635 A allele increased the risk of HIV acquisition and viral load in the Kenyan perinatal cohort [19], whereas in children of European ancestry no significant association with the risk of mother-to-child transmission of HIV infection was found [25]. Among African descendants, the 1635 AA genotype was also associated with higher susceptibility to HIV and hepatitis C virus co-infection [24]. Moreover, another *TLR9* SNP, 1486C/T was also weakly associated with the lower CD4^+^ T cell count [8]. A low average CD4^+^ T cell count, higher HIV load, and a higher probability of disease progression were associated with the 1635 AA genotype [26]. A significant correlation between the 1635 GG genotype and low average CD4^+^ T cell count during the viremic period in HIV-infected patients was found in the second study [27]. The donor genotype at the rs352140 SNP was significant for CMV disease in recipients following HSCT [11]. Clinical evidence has also demonstrated that the donor homozygous recessive genotype of the *TLR9* 1635A/G SNP was associated with a higher incidence of CMV reactivation and yielded a protective effect against acute graft versus host disease in allo-HSCT recipients [12]. Ito et al. [40] reported that the *TLR9* −1486T/C polymorphism was not associated with Vogt-Koyanagi-Harada disease, an autoimmune disorder in which the cytomegalovirus antigen has been hypothesized as a possible triggering factor for the disease. The same SNP has also been found to be significantly associated with bacterial vaginosis among HIV-infected women [28] and bronchiolitis obliterans syndrome in lung transplant recipients [45].

The function of *TLR9* SNPs in host antiviral defense and development of the CMV infection remains unknown. The 2848C/T SNP occurs in exon 2 of the *TLR9* gene but does not lead to any amino acid change (Pro545Pro), while −1237T/C SNP is located within the putative promoter region of the *TLR9* gene and also does not induce an amino acid change. The 1486C/T and 1174G/A polymorphisms are located in the promoter region and intron 1, respectively. Several findings suggest that all polymorphisms can potentially affect *TLR9* expression [31,46,47]. It was found that the 1635 G allele is associated with lower *TLR9* expression, as well as the combination of the C allele at position −1486 with a G allele at position 1174 can downregulate *TLR9* expression [46]. Moreover, Kikuchi et al. [47] reported that the 2848 GG genotype reduced *TLR9* expression at the transcriptional level. Therefore, we speculate that the genetic variation of *TLR9* that downregulates TLR9 expression could reduce the function of the innate immune response against viral infection. We suppose that the other SNPs in linkage disequilibrium might also be responsible for the functional effect of the individual SNPs that were examined.

This study has some limitations. First, our study is limited by its small sample size of HIV/CMV co-infected volunteers. A second limitation is the lack of a control group consisting of age- and sex-matched immunocompetent subjects with CMV DNAemia. However, among immunocompetent individuals, an overt CMV infection is rare, as viral replication is controlled effectively by the immune system. Another limitation of the study includes the fact that HIV-infected patients with CMV co-infection received ART that may have influenced immune responses. Despite several limitations of this study, our data suggest that active CMV infection in HIV-infected individuals may be associated with the *TLR9* 2848C/T polymorphism. To determine whether this association occurs only in the HIV/CMV-infected group or also in other people, the authors plan to evaluate this association in immunocompromised patients not infected with HIV. Further studies using larger patient groups are needed to confirm our results. Moreover, we suggest that functional and mechanistic studies should be conducted to further understand the molecular mechanisms underlying our observations.

## 5. Conclusions

Our study demonstrated significant differences in the rate of occurrence of genetic variants of the *TLR9* gene in HIV/CMV co-infected patients. We found that the 2848 CT and TT genotypes seem to be more frequent in HIV/CMV-infected patients with CMV viremia than in HIV/CMV co-infected subjects without CMV DNAemia. HIV/CMV-positive subjects that are homozygous or heterozygous carriers of the 2848C/T SNP seem to develop a higher CMV load. We suggest that genetic polymorphism may negatively regulate TLR9 signaling and contribute to reduced efficiency of antiviral response, resulting in CMV replication. These findings provide insight into the role of host *TLR9* polymorphism in the frequency of CMV infection among HIV-infected subjects.

## Figures and Tables

**Table 1:** Demographic, clinical, and virological characteristics of the HIV/CMV co-infected adult patients (*n* = 205).

Variables	Results
Age (median and range, years)	37.2 (17–72 years)
Sex, *n* (%)	
female	46/205 (22.4)
male	159/205 (77.6)
CD4 count, *n* (%)	
≤200 cells/mm^3^	43/205 (21.0)
>200 cells/mm^3^	162/205 (79.0)
CD4 nadir, *n* (%)	
≤200 cells/mm^3^	135/205 (65.9)
>200 cells/mm^3^	70/205 (34.1)
CMV IgG, *n* (%)	
positive	192/205 (93.7)
negative	13/205 (6.3)
HBsAg positive, *n* (%)	34/205 (16.6)
anti HCV positive, *n* (%)	54/205 (26.3)
HIV RNA load, *n* (%)	
<20 HIV RNA copies/mL	170/205 (82.9)
>20 HIV RNA copies/mL	35/205 (17.1)
Self-reported HIV-1 transmission route	
MSM	107/205 (52.2)
HET	44/205 (21.5)
MSM/HET	5/205 (2.4)
IDU	49/205 (23.9)

*n*, number of subjects (%); HBsAg, surface antigen of the hepatitis B virus; HCV, hepatitis C virus; MSM, sex between men; HET, sex between women and men; IDU, injecting drug use.

**Table 2:** Genotype and allele frequencies of the *TLR9* SNPs in HIV/CMV co-infected patients.

*TLR9* SNP	Genotype/Allele	Patients with CMV DNAemia, *n* (%)	Patients without CMV DNAemia, *n* (%)	*p*-Value
−1237T/C	TT	117 (96.7)	82 (97.6)	1.000
(rs5743836)	TC	4 (3.3)	2 (2.4)	1.000
	T	238 (98.3)	166 (98.8)	1.000
	C	4 (1.7)	2 (1.2)	1.000
−1486T/C	TT	31 (25.6)	28 (33.3)	0.273
(rs187084)	TC	57 (47.1)	31 (36.9)	0.155
	CC	33 (27.3)	25 (29.8)	0.753
	T	119 (49.2)	87 (51.8)	0.617
	C	123 (50.8)	81 (48.2)	0.617
1174G/A	GG	49 (40.5)	34 (40.5)	1.000
(rs352139)	GA	62 (51.2)	40 (47.6)	0.671
	AA	10 (8.3)	10 (11.9)	0.474
	G	160 (66.1)	108 (64.3)	0.752
	A	82 (33.9)	60 (35.7)	0.752
2848C/T	CC	29 (24.0)	36 (42.8)	0.006
(rs352140)	CT	58 (47.9)	33 (39.3)	0.254
	TT	34 (28.1)	15 (17.9)	0.099
	C	116 (47.9)	105 (62.5)	0.005
	T	126 (52.1)	63 (37.5)	0.005

*n*, number of examined subjects (%); *p*, Fisher’s exact test.

**Table 3:** Associations between target TLR9 polymorphisms and HIV/CMV co-infection.

TLR9	Model	Genotype	Genotype Frequencies; n (%) ^1^		Unadjusted		Adjusted ^2^	
SNPs			Patients with CMV DNAemia	Patients without CMV DNAemia	OR (95% CI)	*p*-Value	OR (95% CI)	***p*-Value**
−1237T/C	-	TT	117 (96.7)	82 (97.6)	1.00	0.7	1.00	1
		TC	4 (3.3)	2 (2.4)	1.40 (0.25–7.83)		1.00 (0.17–5.90)	
1174G/A	codominant	GG	49 (40.5)	34 (40.5)	1.00	0.67	1.00	0.44
		GA	62 (51.2)	40 (47.6)	1.08 (0.60–1.94)		1.04 (0.55–1.97)	
		AA	10 (8.3)	10 (11.9)	0.69 (0.26–1.85)		0.54 (0.19–1.51)	
	dominant	GG	49 (40.5)	34 (40.5)	1.00	1	1.00	0.79
		GA-GG	72 (59.5)	50 (59.5)	1.00 (0.57–1.76)		0.92 (0.50–1.69)	
	recessive	GG-GA	111 (91.7)	74 (88.1)	1.00	0.39	1.00	0.2
		AA	10 (8.3)	10 (11.9)	0.67 (0.26–1.68)		0.53 (0.20–1.40)	
	overdominant	GG-AA	59 (48.8)	44 (52.4)	1.00	0.61	1.00	0.6
		GA	62 (51.2)	40 (47.6)	1.16 (0.66–2.02)		1.17 (0.64–2.14)	
2848C/T	codominant	CC	29 (24.0)	36 (42.8)	1.00	0.014	1.00	0.058
		CT	58 (47.9)	33 (39.3)	2.18 (1.14–4.18)		1.88 (0.89–3.95)	
		TT	34 (28.1)	15 (17.9)	2.81 (1.29–6.14)		2.69 (1.15–6.25)	
	dominant	CC	29 (24.0)	36 (42.8)	1.00	0.004	1.00	0.027
		CT-TT	92 (76.0)	48 (57.2)	2.38 (1.30–4.34)		2.15 (1.09–4.26)	
	recessive	CC-CT	87 (71.9)	69 (82.1)	1.00	0.087	1.00	0.089
		TT	34 (28.1)	15 (17.9)	1.80 (0.91–3.57)		1.86 (0.90–3.83)	
	overdominant	CC-TT	63 (52.1)	51 (60.7)	1.00	0.22	1.00	0.61
		CT	58 (47.9)	33 (39.3)	1.42 (0.81–2.50)		1.18 (0.63–2.20)	

^1^ Values are the number of examined subjects (%); ^2^ Adjusted analysis was carried out for age, sex, HIV RNA load, and CD4^+^ count in whole-blood samples; OR: odds ratio; 95% CI: 95% confidence interval; *p*, logistic regression model, chi-square test. The *p*-value after Bonferroni correction for multiple testing (*p^c^*) is 0.017 (raw *p*-value/3).

## Data Availability

The data generated during the current study are available from the corresponding author on reasonable request.
